# Radiographic Diagnosis of a Central Giant Cell Granuloma Using Advanced Imaging: Cone Beam Computed Tomography

**DOI:** 10.7759/cureus.2735

**Published:** 2018-06-05

**Authors:** Ahmed Z Abdelkarim, Shaimaa M Abu el Sadat, Milda Chmieliauskaite, Ali Z Syed

**Affiliations:** 1 Department of Anatomy, Biochemistry & Physiology, University of Hawaii School of Medicine, Honolulu, USA; 2 Division of Oral Radiology, Department of Oral Medicine, Oral Diagnosis and Oral Radiology,, Faculty of Dentistry, Ain-Shams University, Cairo, Egypt, Cairo, EGY; 3 Department of Oral Medicine and Diagnostic Sciences, CWRU School of Dental Medicine, Cleveland, USA

**Keywords:** advanced imaging, central giant cell granuloma, cbct, mandible, image interpretation, diagnostic imaging, cone-beam computed tomography

## Abstract

Central giant cell granuloma (CGCG) is a benign non-neoplastic, proliferative intraosseous lesion of the jaw with an unknown etiology often diagnosed during the first two decades of life. The true nature of this lesion is controversial and remains elusive. Here, we report a case of central giant cell granuloma, diagnosed using cone-beam computed tomography (CBCT). A 21-year-old female presented to the clinic complaining of a painless swelling involving the right side of the mandible that had started one year previously. A CBCT scan revealed a well-defined, multilocular radiolucent lesion on the right side of the mandible extending from the molar region to the ramus with wispy septations. Wispy septations and undulating borders are some of the characteristic radiographic features of a central giant cell granuloma. The patient underwent an excisional biopsy. The biopsy revealed multinucleated giant cells in a fibrous stroma confirming our radiographic diagnosis of a central giant cell lesion.

## Introduction

The World Health Organization (WHO) classifies central giant cell granuloma (CGCG) as an intraosseous lesion consisting of fibrous cellular tissue that contains multiple foci of hemorrhage, aggregations of multinucleated giant cells, and occasionally trabeculae of woven bone with an incidence rate of 1.1 per million population per year [1-2]. The CGCG was first described by Jaffe in 1953 as a giant-cell reparative granuloma of the jaw bones [3]. Although its pathogenesis and etiology are unknown, its histological and clinical behavior has been studied in detail in the literature [4-5]. CGCG occurs more frequently in females than in males and is more often located in the anterior mandible, mostly crossing the midline, than in the maxilla with patients’ ages ranging from 10 to 25 years old [6]. Its presence in the mandibular body area (the entire ramus, condyle, and coronoid) leads to a therapeutic challenge for maxillofacial surgeons [6].

In most cases, the lesion presents clinically as a painless, slow-growing swelling of the jaw. Pain and sensory disturbances are rare. Intraoral examination usually reveals swelling with a bluish-brown hue that can be observed in some cases. The displacement of teeth occurs frequently and can lead to a malocclusion [6]. Radiological findings of CGCG are diverse, ranging from a small unilocular lesion to large multilocular lesions with the displacement of teeth and tooth germs, root resorption, and cortical perforation [6].

Cone-beam computed tomography (CBCT) has gained immense popularity in maxillofacial imaging because of its high resolution and low radiation dose and is extensively used as a diagnostic imaging modality [7-9]. Here, we report a case of CGCG in the mandible with emphasis on the radiological findings using CBCT imaging.

## Case presentation

A 21-year-old female presented to the Case Western Reserve University School of Dental Medicine Clinic in Cleveland, Ohio. Her chief complaint was a slowly growing painless swelling involving the right side of the mandible that had started one year previously. Her past medical history revealed no previous surgeries or diseases. Her vital signs were recorded as 120/80 mmHg blood pressure, a pulse of 103 beats per minute (bpm), 15 respirations per minute, height - 5.1 ft, weight - 165 lb, and a calculated body mass index (BMI) of 23.71. No other medical conditions were identified, and the patient did not report taking any medications. Clinical examination revealed an intraoral swelling involving the posterior part of the body of the mandible and extending to the ramus on the right side. On palpation, the lesion was hard in consistency, except for select areas which exhibited a softer texture.

**Figure 1:**
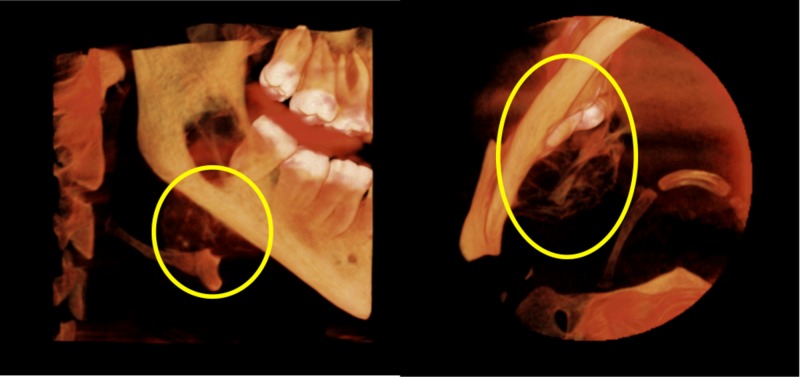
Volume rendering showing medial extension of the lesion (yellow circles)

The patient was referred to a private dental imaging center for a CBCT scan to evaluate the extent of the lesion. A board-certified oral and maxillofacial radiologist performed the radiographic interpretation of the CBCT scan. The scan revealed a well-defined radiolucent lesion ranging from the interdental bone in between the second and third right molars and extending to the ramus of the mandible posteriorly in the anteroposterior direction. The lesion extended from the alveolar crest to the inferior border of the mandible in the superior-inferior direction (Figure 1).

**Figure 2:**
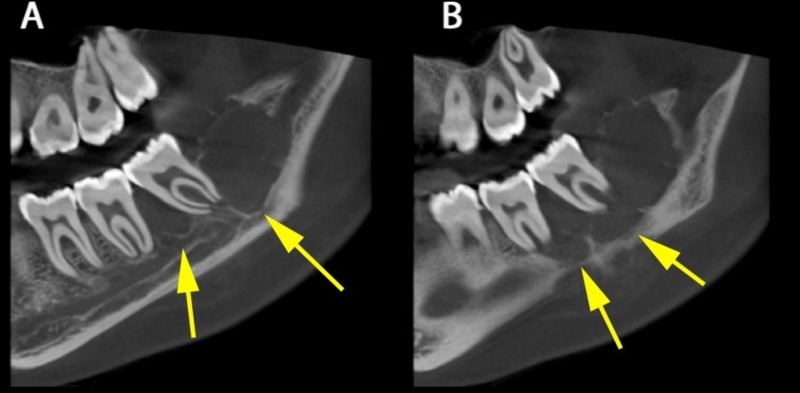
Sagittal images showing undulating border (yellow arrows)

Osteolytic changes of the alveolar crest margin distal to the third molar were noted and displaced the roots of the third molar more distally. The sagittal cut showed that the lesion had a multilocular appearance with an incomplete internal septal structure, demonstrating wispy-like septations. The inferior border of the mandible showed some resorption with undulating borders (Figure 2). The lesion showed expansion of the alveolar crest. In axial cuts, the lesion involved the ramus of the mandible, and expansion and thinning of the inner cortical plate were noted.

**Figure 3:**
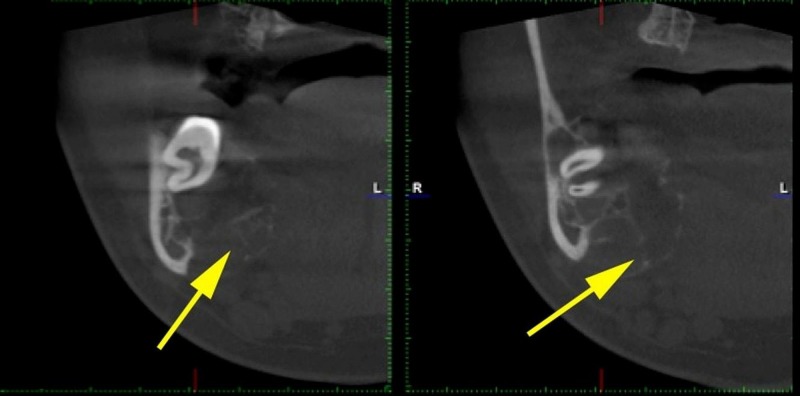
Thin, wispy septations are shown using yellow arrows

Extending more medially, the borders of the lesion showed fine wispy-like septations with undulating borders (Figure 3). Coronal slices demonstrated the resorption of the outer cortical boundary, a multilocular appearance, an expansible nature, and thinning and resorption of the inner cortical boundary. The presence of an intact (albeit thinning) border, wispy septations, and expansion led to a provisional diagnosis of an aggressive benign tumor. Central giant cell granuloma, ameloblastoma, and keratocystic odontogenic tumor (KOT) were considered in the differential. However, the presence of fine, wispy septations and undulating borders favored a CGCG diagnosis. 

An incisional biopsy was performed, and the histopathological report for the lesion revealed a giant cell reparative granuloma formed of proliferating spindle cells, multinucleated giant cells, extravasated red blood cells (RBCs), and mononuclear cells. No specific inflammatory granuloma or significant nuclear anaplasia and mitosis or other evidence of malignancy were noticed. This information, along with clinical and radiological features, was suggestive for CGCG and hyperparathyroidism (because of similar histopathological features). Laboratory investigations showed that the parathyroid hormone, alkaline phosphates, and calcium levels were all within normal limits. This excluded hyperparathyroidism as a possible diagnosis. The patient underwent surgical resection of the lesion. The surgery and recovery were uneventful.   

## Discussion

This case report describes the unusual location of a central giant cell granuloma (CGCG). In this case; the patient was 21 years old at the time of the first examination and the lesion was noted in the posterior aspect. A CGCG of the jaw is more commonly found in the anterior mandible than in the maxilla and found in females more often than in males. These lesions occur before the age of 30 [10].

CGCG has a variable clinical behavior. It may show an aggressive rapidly growing expansive lesion or be small, asymptomatic, and slow-growing [11]. Clinical findings, in this case, included an asymptomatic, slowly increasing swelling of the mandible that extended to the ramus on the right side.

Conventional two-dimensional radiography is generally the first imaging modality performed in clinical practice. However, it provides limited information regarding the cortical integrity, size, and extension of the lesion, and thus imaging providing more in-depth detail, such as three dimensional (3D) imaging, is preferred [9, 12]. Cortical disruptions and soft tissue involvement can be better appreciated in 3D imaging, such as computed tomography (CT) images, which can also help define the extent of the lesion and its internal structure for diagnostic and baseline [10]. The radiographic examination, in this case, was done using CBCT. CBCT was used because of its low dose to the patient in comparison with CT and is the preferred imaging modality in maxillofacial imaging [12]. The histological and radiographic appearance of CGCG is similar to brown tumors observed in hyperparathyroidism [13-14]. Age and laboratory investigations are required to rule out hyperparathyroidism.

## Conclusions

CGCG is a benign, non-neoplastic proliferative intraosseous lesion of the jaw with an unknown etiology, which is diagnosed during the first two decades of life. CBCT offers more information about lesion extension and cortical wall integrity and aids in diagnosis.

## References

[1] Ebrahimi H, Yazdani J, Pourshahidi S (2008). Central giant cell granuloma of the posterior maxilla: a case report. J Dent Res Dent Clin Dent Prospects.

[2] Geetha N, Pattathan RK, Shivakumar H, Upasi A (2011). Fibro-osseous lesions vs. central giant cell granuloma: a hybrid lesion. Ann Maxillofac Surg.

[3] Garg P, Jain J, De N, Chatterjee K (2017). A central giant cell granuloma in posterior part of maxilla - A case report. Int J Surg Case Rep.

[4] de Lange J, van den Akker HP (2005). Clinical and radiological features of central giant-cell lesions of the jaw. Oral Surg Oral Med Oral Pathol Oral Radiol Endod.

[5] Liu B, Yu SF, Li TJ (2003). Multinucleated giant cells in various forms of giant cell containing lesions of the jaws express features of osteoclasts. J Oral Pathol Med.

[6] de Lange J, Van den Akker HP, van den Berg H (2007). Central giant cell granuloma of the jaw: a review of the literature with emphasis on therapy options. Oral Surg Oral Med Oral Pathol Oral Radiol Endod.

[7] Syed AZ, Zahedpasha S, Rathore SA, Mupparapu M (2016). Evaluation of canalis basilaris medianus using cone-beam computed tomography. Imaging Sci Dent.

[8] Syed AZ, Hawkins A, Alluri LS (2017). Rare finding of Eustachian tube calcifications with cone-beam computed tomography. Imaging Sci Dent.

[9] Scarfe WC, Farman AG (2008). What is cone-beam CT and how does it work?. Dent Clin North Am.

[10] Eisenbud L, Stern M, Rothberg M, Sachs SA (1988). Central giant cell granuloma of the jaws: experiences in the management of thirty-seven cases. J Oral Maxillofac Surg.

[11] Motamedi MH, Talesh KT, Jafari SM, Khalifeh S (2010). Peripheral and central giant cell granulomas of the jaws: a retrospective study and surgical management. Gen Dent.

[12] Syed AZ, Sin C, Rios R, Mupparapu M (2016). Incidental occurrence of an unusually large mastoid foramen on cone-beam computed tomography and review of the literature. Imaging Sci Dent.

[13] Chrcanovic BR, Gomes CC, Gomez RS (2018). Peripheral giant cell granuloma: an updated analysis of 2824 cases reported in the literature. J Oral Pathol Med.

[14] White SC, Pharoah Pharoah, MJ MJ (2014). Oral Radiology Principles and Interpretation, Edition 7. http://books.google.com/books?id=WQEyAgAAQBAJ&printsec=frontcover&dq=Oral+Radiology+Principles+and+Interpretation&hl=en&sa=X&ved=0ahUKEwi5porn5rrbAhXkMn0KHahFC58Q6AEIJzAA#v=onepage&q=Oral%20Radiology%20Principles%20and%20Interpretation&f=false.

